# A New Hyperchaotic System-Based Design for Efficient Bijective Substitution-Boxes

**DOI:** 10.3390/e20070525

**Published:** 2018-07-12

**Authors:** Eesa Al Solami, Musheer Ahmad, Christos Volos, Mohammad Najam Doja, Mirza Mohd Sufyan Beg

**Affiliations:** 1 Department of Information Technology, University of Jeddah, Jeddah 21589, Saudi Arabia; 2 Department of Computer Engineering, Jamia Millia Islamia, New Delhi 110025, India; 3 Department of Physics, Aristotle University of Thessaloniki, 54124 Thessaloniki, Greece; 4 Department of Computer Engineering, Aligarh Muslim University, Aligarh 202002, India

**Keywords:** substitution-box, bijective, 5-D hyperchaotic system, batch-generation, small-sized S-boxes

## Abstract

In this paper, we present a novel method to construct cryptographically strong bijective substitution-boxes based on the complicated dynamics of a new hyperchaotic system. The new hyperchaotic system was found to have good characteristics when compared with other systems utilized for S-box construction. The performance assessment of the proposed S-box method was carried out based on criteria, such as high nonlinearity, a good avalanche effect, bit-independent criteria, and low differential uniformity. The proposed method was also analyzed for the batch-generation of 8 × 8 S-boxes. The analyses found that through a proposed purely chaos-based method, an 8 × 8 S-box with a maximum average high nonlinearity of 108.5, or S-boxes with differential uniformity as low as 8, can be retrieved. Moreover, small-sized S-boxes with high nonlinearity and low differential uniformity are also obtainable. A performance comparison of the anticipated method with recent S-box proposals proved its dominance and effectiveness for a strong bijective S-box construction.

## 1. Introduction

Recent advancements in cloud computing, smart devices, social media, etc., for communication have substantially raised the amount of users’ private data. Consequently, the issue of ensuring and maintaining end to end confidentiality of sensitive data has become more prominent than before. To provide data secrecy for storage and communication, block cryptosystems have been playing a crucial role in the past few decades. In modern block ciphers, cryptographically potent S-boxes are meant to serve this purpose to meet Shannon’s requirement of confusion. S-boxes are cornerstone components in many substitution-permutation (S-P) networks or Fiestal networks-based block cryptosystems, such as the famous data encryption standard (DES), Blowfish, advanced encryption standard (AES), Anubis, PRESENT, etc. [1]. According to C.E. Shannon, the confusion establishes a correlation between a secret key and the encrypted text such that it is as complicated and intricate as possible [2]. To achieve a strong confusion, applying complicated and highly nonlinear transformations is demanded. The security of block ciphers directly relies on the strength of the S-boxes employed. Therefore, designing methods that are credible to yield strong S-boxes have drawn the attention of security experts and researchers worldwide [3]. 

An *n* × *n* substitution-box takes a small block of *n* bits and transforms it to an output that is *n* bits long. It acts as a nonlinear mapping *S*: {0, 1}*^n^* → {0, 1}*^n^* [4], and can be viewed as a Boolean vector that consists of *n* Boolean functions, each in *n*-variable as *S*: *g_n_*(*x*)*g_n_*_−1_(*x*)……*g*_1_(*x*), where each *g_i_*(*x*) is a function from {0, 1}*^n^* to {0, 1}, and *g_i_*(*a*) = *b_i_* for *i* = 1, 2, ……, *n* [5]. A Boolean function *g_i_* is balanced if its outputs have an equal distribution of 0’s and 1’s. A bijective S-box of size *n* × *n*, involving balanced component functions, has distinct pre-images in the range [0, 2*^n^* − 1]. The bijectivity of the S-box is verified through the satisfaction of $hwt\left(\sum_{i=1}^{n}a_{i}g_{i}\right)=2^{n-1}$ [5], where, *hwt*() denotes the hamming weight, $a_{i}\in \{0,1\}$, and (*a*_1_, *a*_2_, …, *a*_8_) ≠ (0, 0, …, 0). Bijective S-boxes of different sizes have significance in many cryptographic primitives and S-P network based ciphers. They have been utilized in many popular ciphers such as AES, PRESENT, ARIA, KASUMI, hash function KECCAK, etc. [1]. Recently, the application of S-boxes has also been investigated for the design of image encryption, video encryption, watermarking, pseudo-random sequence design, etc.

Chaos is a ubiquitous phenomenon in nature that is being widely utilized for numerous applications in various fields of study, such as engineering, mathematics, physics, biology, and so on [6]. It has deterministic and noise-like behavior, and it is present in nonlinear dynamical systems. The generated sequences from chaotic systems are extremely sensitive to their initial conditions, have a long periodicity, ergodicity, and spread spectrum [7]. These features of chaotic systems have a close correlation with properties of cryptography. Therefore, they have been a preferred choice for designing security primitives and cryptosystems in chaos-based cryptography for long time [8]. 

A dynamical system is said to have a high sensitivity to initial conditions and parameters provided that it has positive Lyapunov exponent(s). The Lyapunov exponent (*LE*) of a nonlinear dynamical system refers to the pace of separation of infinitesimally close trajectories. Mathematically, it is defined as:

(1) $$LE_{i}=\underset{t\to \infty }{lim}\frac{1}{t}\log_{2}\left[\frac{p_{i}(t)}{p_{i}(0)}\right]$$

where *p_i_*(*t*) denotes the length of the respective ellipsoidal principal axis. An *n*-D system has *n* number of Lyapunov exponents. Presence of only one positive exponent indicates the existence of chaotic behavior in a system. Whereas, if it has at least two positive LEs, then the system is hyperchaotic in nature [9]. Simple-structured and one positive *LE* dynamical systems tend to possess weak security due to a common correlation that allows it to be cryptanalyzed [10]. Compared to chaotic systems, hyperchaotic systems tend to show more complex dynamics. The minimum dimension for a dynamic system to exhibit hyperchaotic nature should be 4-D. The research has matured in the direction of designing higher dimensional hyperchaotic systems and the present focus is on designing 5-D systems with better dynamics and characteristics [10,11,12,13,14,15]. Hyperchaotic systems have found applications in the area of security for realizing cryptosystems [16], hash functions [17], secure communication [18], S-boxes [19], etc.

In the recent past, a number of high-dimensional chaotic systems have been utilized to design methods for S-box generation. Of late, Islam et al. investigated a 4-D hyperchaotic system where two pseudo-random 8-bit integer sequences were produced, which gave rise to an S-box after a two-position swap operation [19]. In Reference [20], Özkaynak adopted a Lorenz chaotic system by sampling the system trajectory after gaps of time steps. A coding table was formed, which resulted in an S-box after some shifting operations. The method of Khan et al. [21] explored multiple systems, namely 3D Lorenz and Rössler chaotic systems, to randomly generate all possible elements of an 8 × 8 S-box. The same author investigated a fractional order Rössler chaotic system and suggested a simple method to synthesise S-boxes in Reference [22]. It was noted that their design is consistent for secure communication. Liu et al. [23] applied a 3-D four-wing chaotic system to generate S-boxes with a good performance. In Reference [24], another simple method was developed for S-boxes based on a fractional-order Chen chaotic system. Cavusoglu et al. [25] scaled the 3-D Zhongtang chaotic system to design a random number generator (RNG) for constructing 8 × 8 S-boxes based on this RNG.

In this paper, a new high-dimensional hyperchaotic system is explored for generating strong S-boxes. The proposed hyperchaos-based S-box construction method is capable of synthesizing strong bijective S-boxes, and was found to possess excellent cryptographic strength when compared with some recent S-box proposals. The main contributions of this paper include the following:

- A 5-D hyperchaotic system is proposed that has twelve terms, seven system parameters, and three cubic nonlinear product terms.
- Based on the new hyperchaotic system, an S-boxes construction method is proposed to synthesize efficient 8 × 8 S-boxes, and these are compared with recent methods.
- Performance of the same method is also investigated for a generation of small-sized bijective S-boxes ranging from size 4 × 4 to 7 × 7, and is compared with some rare and prominent methods.

The remaining content of the paper is as follows. The model of the newly proposed 5-D hyperchaotic system is discussed and analyzed in Section 2. The proposed method for constructing strong S-boxes is presented in Section 3. Performance assessment of proposed method for S-boxes is performed in Section 4 and compared with some of the most recent S-box methods. This section is subsequently followed by conclusions drawn in Section 5. 

## 2. 5-D Hyperchaotic System

Our novel 5-D nonlinear dynamical system is governed by the following state equations:

(2) $$\begin{array}{l} \dot{x}=-c_{1}x+c_{1}y\\ \dot{y}=c_{2}x+c_{2}y+w-xzu\\ \dot{z}=-c_{3}y-c_{4}z-c_{5}u+xyu\\ \dot{u}=-c_{6}u+xyz\\ \dot{w}=-c_{7}x-c_{7}y \end{array}\}$$

where, *c_i_* (1 ≤ *i* ≤ 7) are the system’s seven constants and *x*(0), *y*(0), *z*(0), *u*(0), *w*(0) are the initial conditions that decide its trajectories in phase space. Computation of Lyapunov exponents is performed following the well-known procedure reported in Reference [26]. Interestingly, when parameters are set as *c_i_* = {30, 10, 15.7, 5, 2.5, 4.45, 38.5}, the obtained Lyapunov exponents for system (2) are *LE*_1_ = 4.90182, *LE*_2_ = 0.38463, *LE*_3_ = 0, *LE*_4_ = −15.86286, and *LE*_5_ = −31.90952, thereby confirming the existence of hyperchaos as there are two positive exponents. The proposed 5-D hyperchaotic system (2) holds some positive characteristics, which are as follows:

- It contains three cubic-order nonlinear product terms, which is rare as the order of nonlinear product terms is usually quadratic; this strengthens the system against some parameter identification attacks [27].
- It has a large number of systems parameters, namely seven; these are with five initial conditions that heavily enlarge the secret key space of the respective security primitive to make brute-force attack impractical.
- The value of the largest Lyapunov exponent is 4.90182, which is quite high. This value is substantially higher than 0.9899 [10], 0.0981 [11], 0.0792 [12], 0.4195 [13], and 0.5441 [14] in recent 5-D hyperchaotic systems. A larger positive Lyapunov exponent shows that system trajectories vary more sharply in phase space and this makes the system’s dynamics more complicated by establishing stronger sensitivity to initial conditions.
- It is invariant under coordinate transformation (*x*, *y*, *z*, *u*, *w*) → (−*x*, −*y*, −*z*, −*u*, −*w*); that is, the symmetry persists for all system variables.
- The Kaplan-Yorke (Lyapunov) dimension *D_KY_* for any dynamical system is defined as [28]:
  $$D_{KY}=j+\frac{1}{\left|LE_{j+1}\right|}\sum_{i=1}^{j}LE_{i}$$
  where, *j* is the largest integer for which $\sum_{i=1}^{j}LE_{i}\geq 0$ and $\sum_{i=1}^{j+1}LE_{i}<0$. The Lyapunov dimension *D_KY_* for the hyperchaotic system (2) is *D_KY_* = 3.334, which indicates that the Lyapunov dimension of our system is fractional.
- The vector field of our system (2) has negative divergence as:
  (3) $$\nabla V=\frac{\partial \dot{x}}{x}+\frac{\partial \dot{y}}{y}+\frac{\partial \dot{z}}{z}+\frac{\partial \dot{u}}{u}+\frac{\partial \dot{w}}{w}=-c_{1}+c_{2}-c_{4}-c_{6}<0$$
  This indicates that the system (2) is dissipative in nature, with an exponential contraction rate of $\frac{dV}{dt}=e^{-(c_{1}-c_{2}+c_{4}+c_{6})t}$. Dissipation is needed to attract trajectories in the system’s phase space.

**Figure 1:**
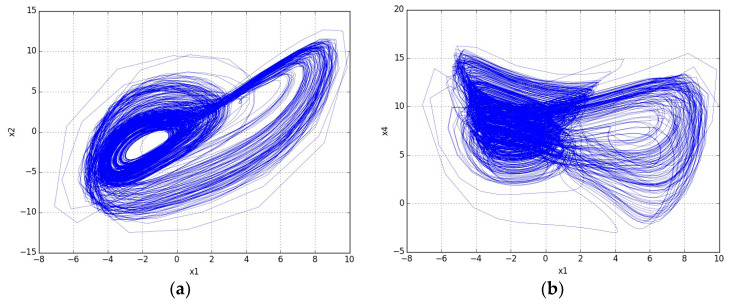

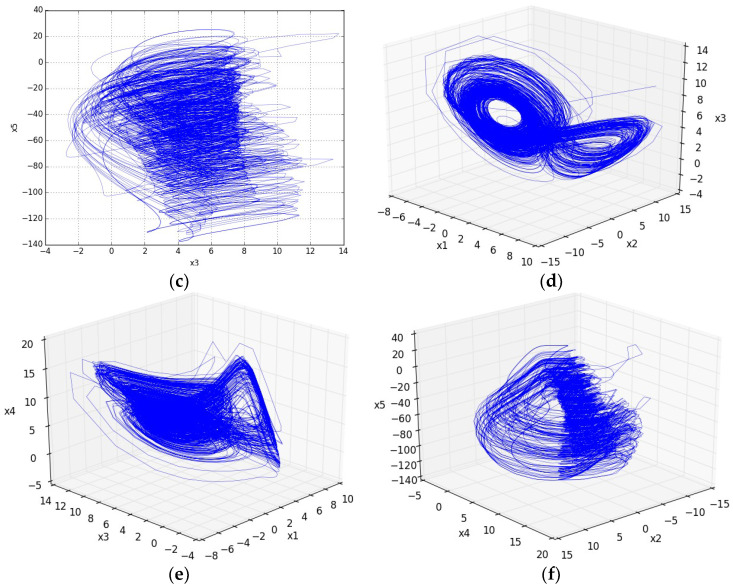
Phase portraits of the 5-D hyperchaotic system (2) (**a**) projection on the *x*-*y* plane; (**b**) projection on the *x*-*u* plane; (**c**) projection on the *z*-*w* plane; (**d**) 3-D view in *x*-*y*-*z* space; (**e**) 3-D view in *x*-*z*-*u* space; and (**f**) 3-D view in *y*-*u*-*w* space.

The characteristics of the proposed nonlinear system (2) are compared with some high-dimensional chaotic/hyperchaotic systems adopted by researchers to construct S-boxes in Table 1. It is worth noting that unlike our method, in all high dimensional system based S-box methods [19,20,21,22,23,24,25,29], the systems were not modeled by S-box investigators. The comparison table ascertains that system (2) holds excellent characteristics over other adopted systems. Figure 1 displays the phase portraits of the proposed system in various planes and phase spaces. It should be noted that the phase portrait is only an indicative tool for displaying a system’s dynamic behavior. Thus, from the phase portraits, we assume the system’s (2) complex behavior, which is confirmed by the calculation of the Lyapunov exponents for a chosen set of the system’s parameters.

## 3. Proposed Bijective S-Box Generation Method

The method proposed to efficiently search for S-boxes using the new 5-D hyperchaotic system is provided below. This method performs a random search on the basis of the maximization of nonlinearity to find an optimal configuration of the *S_G_* S-box. The proposed method calls the *hyperchaos5D*() routine, which solves system (2) using Runge-Kutta of order 4 with a step size of 0.001, and takes the initial conditions of five state variables to produce the variable’s floating values after *t*_0_ or *τ* iterations. Routine *reverse*() is intended to reverse the input vector. *CreateS*() is used to prepare an S-box candidate using a random input vector, and the *sort*() function performs sorting of the input array in increasing order. The routine *nonlinearity*() is meant to compute the average of the nonlinearities of all component Boolean functions of the input S-box. The details of the nonlinearity metric are discussed in Section 4. The *max*() function returns the largest among all inputs and *index* ∈ [1,5] return the largest value in the input vector.

The proposed method prefers an S-box over previous methods on the basis of nonlinearity of the S-boxes. Since these two are considered to be mainly responsible for strong confusion, nonlinear transformation, and the potential to mitigate differential and linear attacks. S-box *S_G_* is updated as *S_P_* if and only if *S_P_* is no worse than *S_G_* on the grounds of nonlinearity. The target nonlinearities for the bijective S-box of dimension *n* = 4, 5, 6, 7, 8 are 4, 12, 24, 56, 112, respectively [30].

1. Assign initial values to size *n,* parameters *c_i_*, initial conditions for *x*, *y*, *z*, *u*, *w*, *t*_0_, *τ*, *itr_max*   Set *nl_max* = 0, *numel* = 2*^n^*, *len* = *numel*/2, *x*_1_(0) = *x*, *x*_2_(0) = *y*, *x*_3_(0) = *z*, *x*_4_(0) = *u*, *x*_5_(0) = *w*2.  Take *S_P_*, *S_G_* as two empty look-up tables(LUTs) and five empty arrays *X_j_*, each of size *numel*3. Iterate system (2) *t*_0_ times to die out the transient effect and discard *x_j_* except the last:    [*x*_1_, *x*_2_, *x*_3_, *x*_4_, *x*_5_] = *hyperchaos5D*(*x*_1_(0), *x*_2_(0), *x*_3_(0), *x*_4_(0), *x*_5_(0), *t*_0_)    *x*_1_(0) = *x*_1_, *x*_2_(0) = *x*_2_, *x*_3_(0) = *x*_3_, *x*_4_(0) = *x*_4_*, x*_5_(0) = *x*_5_4. Generate the lower halves of the random arrays *X_j_* as:    *for k* = 1 to *len*    [*x*_1_, *x*_2_, *x*_3_, *x*_4_, *x*_5_] = *hyperchaos5D*(*x*_1_(0), *x*_2_(0), *x*_3_(0), *x*_4_(0), *x*_5_(0), *τ*)    *x_j_*(0) *=* (*x_j_* × 10,000) *− floor*(*x_j_ ×* 10,000)    *X_j_*(*k* + *len*) = *x_j_*(0)    *end*5. Generate new higher halves of the random arrays *X_j_* as:    *X_j_* = *reverse*(*X_j_*)    *for k* = 1 to *len*    [*x*_1_, *x*_2_, *x*_3_, *x*_4_, *x*_5_] = *hyperchaos5D*(*x*_1_(0), *x*_2_(0), *x*_3_(0), *x*_4_(0), *x*_5_(0), *τ*)    *x_j_*(0) *=* (*x_j_* × 10,000) *− floor*(*x_j_ ×* 10,000)    *X_j_*(*k* + *len*) = *x_j_*(0)    *end*6. Create S-box candidates:    *S_j_* = *CreateS*(*X_j_*)7. Compute nonlinearity of candidates *S_j_*:    *nl_j_* = *nonlinearity*(*S_j_*)8. Choose the local best candidate:    [*nl_P_*, *index*] = *max*(*nl*_1_, *nl*_2_, *nl*_3_, *nl*_4_, *nl*_5_)    *S_P_* = *S_index_* // where, *S_index_* = *S_j_* for *j* = *index*9. Update the global best candidate (if required):    *If* (*nl_P_* ≥ *nl_max*)    *S_G_* = *S_P_*    *nl_max* = *nl_P_*    *end*10. Repeat from step 5 for *itr_max* times.11. Declare *S_G_* as the final S-box and display as LUT.

*S* = *CreateS*(*X*)1. *Y* = *sort*(*X*)2. *for k*_1_ = 1 to *numel*3.  *t* = *Y*(*k*_1_)4.  *for k*_2_ = 1 to *numel*5.   if(*t* = = *X*(*k*_2_))6.    *S*(*k*_1_) = *k*_2_ − 17.    *break*8.   *end*9.  *End*10. *end*

## 4. Performance Analysis

For the simulation, the experimental values are initialized as *c_i_* as provided earlier, *x*_1_(0) = 0.8*, x*_2_(0) = 4.9, *x*_3_(0) = 7.6, *x*_4_(0) = 3.7, *x*_5_(0) = 6.5, *t*_0_ = 1000, and *τ* = 2. The secret key includes *c_i_*, *x_j_*(0), *t*_0_, and *τ.* In order to avoid the problem of dynamic degradation, we carried out all floating point computation as per the IEEE-754 floating point standard of double floating point arithmetic. In our working 15-digit precision implementation system, the key space is more than 10^180^ ≈ 2^598^, which quite large enough to resist brute-force attack. The proposed S-box obtained for *n* = 8 is shown in Table 2. It is well-known that an S-box is deemed strong if it satisfies a number of performance criteria. This section deals with the performance analysis of the proposed 8 × 8 S-box against a number of well-accepted criteria, such as bijectivity, nonlinearity, strict avalanche criteria, bits independent criterion, differential uniformity, and linear approximation probability [19,20,21,22,23,24,25,31]. The security strength of the S-box in Table 2 is compared with recent S-boxes.

### 4.1. Bijectiveness

A bijective function is a combination of one-to-one (injective) and onto (surjective) mapping functions. It implies that every element of one set is paired with exactly one element of the other set, and vice versa, with no unpaired elements. This is an important property that is used to test the cryptographic liability of S-boxes. It is verified from S-box LUTs that a proposed S-box satisfies the bijectivity property as it has distinct pre-images in a specified range.

### 4.2. Nonlinearity

In the nonlinearity analysis, the constituent Boolean functions were assessed with reference to the behavior of the input/output bit patterns. The set of all affine functions is used to compare the distance from the given Boolean function. Once the initial distance is determined, the bits in the truth table of the Boolean function were modified to approximate to the closest affine function. The number of modifications required to reach the closest affine functions determined the nonlinearity of the Boolean function. In practice, the nonlinearity of the Boolean function *g* in *n*-variable is measured using Equation (3) through Walsh spectrum [32]:

(4) $$nl(g)=2^{n-1}\left(1-2^{-n}\underset{\omega \in {\left\{0,1\right\}}^{n}}{\max}\left|WS_{g}(\omega )\right|\right)$$

where *WS_g_*(*ω*) is the Walsh spectrum of function *g*, computed as:

$$WS_{g}(\omega )=\sum_{x\in {\left\{0,1\right\}}^{n}}{\left(-1\right)}^{g(x)⊕x.\omega }$$

where, *x*.*ω* refers to a bit-by-bit dot product and *ω* ∈ {0, 1}*^n^*. It is also expressed as the least hamming distance between the set of all non-constant linear combinations of function *g* and set of all affine functions on {0, 1}*^n^* [33]. The best affine and linear approximation attacks [34,35] show the significance of constructing S-boxes with high nonlinearity. The nonlinearity of Boolean functions in the proposed 8 × 8 S-box, provided in Table 3, are found as *nl*(*g*_1_) = 110, *nl*(*g*_2_) = 108, *nl*(*g*_3_) = 110, *nl*(*g*_4_) =106, *nl*(*g*_5_) = 108, *nl*(*g*_6_) = 108, *nl*(*g*_7_) = 110, and *nl*(*g*_8_) = 108, showing that *nl_min_* = 106, *nl_max_* = 110, and *nl_avg_* = 108.5. The nonlinearity values of all component functions were quite high, and larger than or equal to 106. It clearly shows the nonlinearity of the proposed S-box. 

### 4.3. Strict Avalanche Criteria

The idea of a strict avalanche criterion (SAC) is a generalization of the avalanche effect, introduced by Webster and Tavares in 1985 [36], is based on Shannon’s property of diffusion and implies that a little change in input causes a significant effect in the output. According to Webster and Tavares, if a Boolean function satisfies SAC, it means that if we change any one of the input bits, then all the output bits should change with a probability of a half. The SAC can be evaluated through an 8 × 8 dependency matrix by a procedure suggested in Reference [36]. The average of this matrix is referred to as the SAC value. We calculated the dependency matrix for the proposed S-box and this is shown in Table 4. It can be seen that almost all values are somewhat close to 0.5. The average of the dependency matrix is SAC = 0.5017, which is fairly close to the theoretical SAC with an offset of only 0.0017; this shows that the proposed S-box exhibited a good avalanche effect and satisfied the stated criteria well.

### 4.4. Bits Independence Criteria

The bits independent criterion (BIC) manages testing an individual bit at the input of the cipher by playing out the flip operation. It implies that all the avalanche vectors ought to be match pair-wise independent for a given arrangement of vectors produced by complementing a solitary plaintext bit. The avalanche vectors are created by bit patterns generated because of flipping bit(s) at the inputs. It is an attractive property for any cryptographic primitive. The S-box fulfills BIC if the function *g* = *g_i_* xor *g_j_* (*i* ≠ *j*, 1 ≤ *i*, *j* ≤ 8) is highly nonlinear and also satisfies the SAC [32]. Based on this method, BIC for the proposed S-box was verified by computing the nonlinearity and SAC of *g* = *g_i_* xor *g_j_* [37]. The result of BIC for nonlinearity is provided in Table 5 and that of the BIC for the SAC is in Table 6. The average of the BIC-nonlinearity is 104 with a least value of 100 (a commendable score), and the average of the BIC-SAC matrix is 0.5006, which is very close to 0.5. The scores indicate that the proposed S-box is competent enough to satisfy the output bits independence criteria.

### 4.5. Differential Uniformity

The differential uniformity (DU) measure is associated with the change in the output or the differential output observed with respect to a change in input. Its intensity determines the S-box’s ability to resist the differential cryptanalysis framed by Biham and Shamir to break the famous DES block cipher [38]. The differential uniformity of an S-box ensures uniform mapping of the input and output differentials. It denotes the maximum likelihood of generating an output differential *δb* = *b_i_* xor *b_j_* when the input differential is *δa* = *a_i_* xor *a_j_*. Mathematically, it is expressed as [23,38,39]:

(5) $$du_{S}=\underset{\delta a\neq 0,\delta b}{\max}\left(\#\left\{a\in A|S(a)⊕S(a⊕\delta a)=\delta b\right\}\right)$$

where, # denotes cardinality, and *X* is the set of all inputs *x*. The output exclusive XOR score as explained should have equal likelihood for a corresponding input score. As a good S-box design guideline, the maximum differential uniformity has to be kept as small as possible to resist differential cryptanalysis. Following the approach, an input/output XOR distribution matrix, for differential, is obtained for the proposed S-box and is provided in Table 7. The maximum differential uniformity for our S-box was 10, which is the highest value of the differential matrix in Table 7. The count of this highest value in differential table is only 4. This value of DU is compared with some recent S-boxes in Table 8 to show the effectiveness of proposed S-boxes.

### 4.6. Comparison

The comparison is done in Table 8 based on the criteria discussed in previous subsections. The outcomes of the comparisons are as follows:

The nonlinearity strength of the proposed S-box is worth noting as its average value *nl_avg_* of 108.5 was the highest among all S-box methods in Table 8. The *nl_min_* is similar to three S-boxes in References [40,41,50,52], and better than all other S-boxes. Similarly, the *nl_max_* value was comparable to two S-boxes investigated in References [25,33], and larger than the remaining S-boxes. Thus, the proposed S-box provided high nonlinearity, and in turn, strong confusion, and good resistance to linear and affine approximation attacks while transforming input plaintext bits to output bits.

The ideal value for SAC is 0.5, any value closer to this is considered as better than others. According to Table 8, our SAC of 0.5017 is closer to the ideal value and better than the SAC of almost all S-boxes in Table 8. However, all SAC scores in Table 8 were more or less close to 0.5 with almost negligible offsets. The proposed S-box satisfied the SAC criteria very well, and marginally outperformed most of the other S-boxes.

According to the BIC test, referring to Table 5 and Table 6 for our S-box, the average of the BIC-nonlinearity is 104, which is higher than the value reported in References [20,22,24,25,29,39,40,42,44,45,46,48,49,51], and the BIC-SAC is 0.5026, which is again quite close to 0.5 and better than in References [23,24,25,40,42,44,45,46,47,48,50,51]. The BIC performance of the proposed S-box for nonlinearity and SAC is satisfactory.

The S-box should have adequacy to thwart differential cryptanalysis as practiced by Biham and Shamir [38]. It is well accepted that an S-box having a lower DU offers more resistance to this cryptanalysis. It is worth noting that the DU of the proposed S-box was only 10, which is similar to the DU of the S-boxes investigated in References [19,20,23,24,25,39,40,43,49,50,52], and better than the DU of the S-boxes in References [21,22,29,33,41,42,43,44,45,46,47,48,51]. This means there is an excellent fulfillment of our method on the DU criteria of strong S-boxes.

### 4.7. Analysis of the Batch-Generation of 8 × 8 S-Boxes

**Figure 2:**
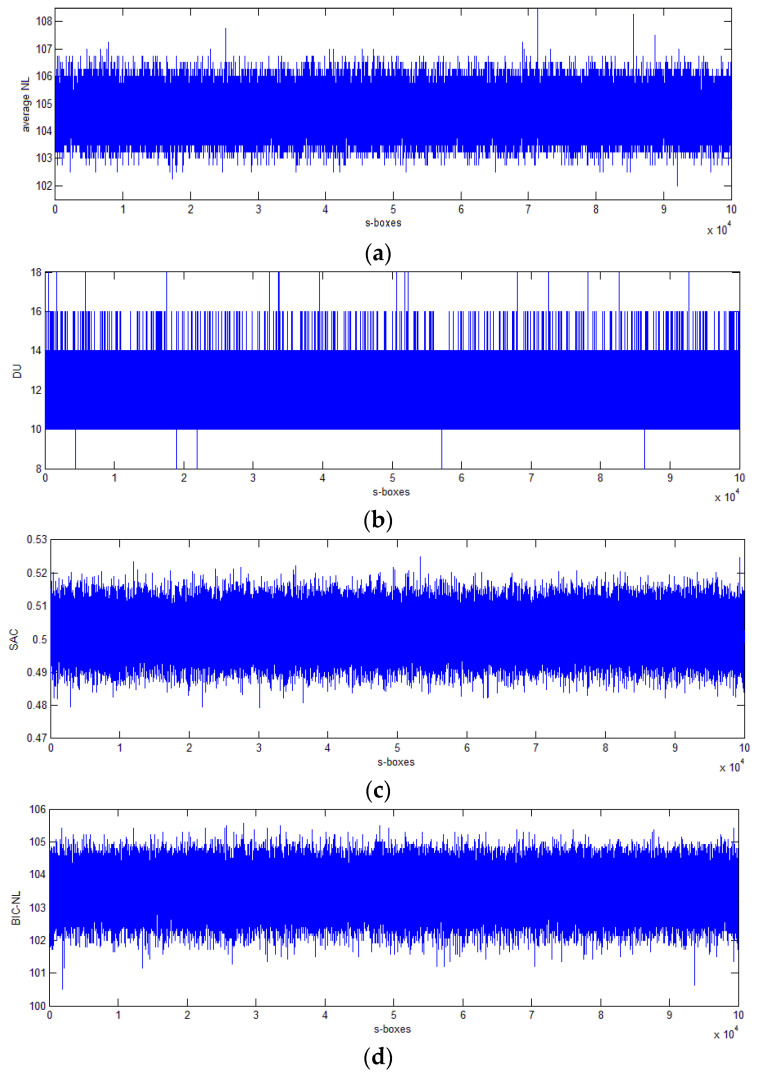

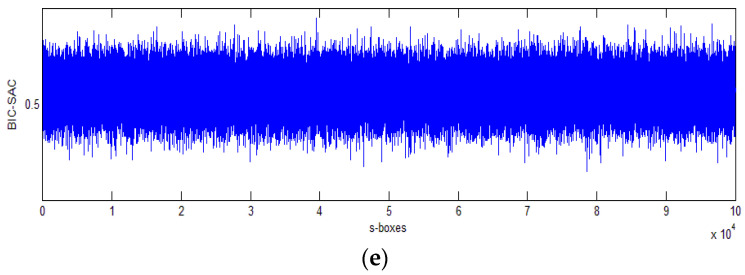
Performance of generation of 100,000 8 × 8 S-boxes for (**a**) average nonlinearity, (**b**) maximum differential uniformity, (**c**) SAC, (**d**) average BIC-nonlinearity, and (**e**) BIC-SAC.

This subsection deals with the performance analysis of all intermediate 8 × 8 *S_P_* S-boxes obtained while running the proposed method for *itr_max* = 100,000. The features of these S-box structures, such as average nonlinearity, differential uniformity, SAC, BIC-nonlinearity, and BIC-SAC, are shown graphically in Figure 2. The statistics of these S-boxes for worst, best, average cases, and different conditions are reported in Table 9 and Table 10. 

Performance statistics showed that the batch-generation capability of the proposed method was excellent. Reason being, the worst, best, and average nonlinearities of all intermediate S-boxes are 102, 108.5, and 104.665, respectively, which is far better than the respective statistics of 99.25, 106.75, and 103.55, respectively, of S-box structures by Özkaynak’s recent method [40]. This means that all generated 100,000 S-boxes had an average nonlinearity equal to or above 102. The proposed method generated 90.932% S-boxes with average nonlinearity higher than or equal to 104, and 53.467% had a nonlinearity greater than or equal to 105. As far as differential uniformity is concerned, there were 40.871% S-boxes having a DU less of than or equal to 10. The worst DU was 18, and only 16 such S-boxes exists; the best achievable DU with the proposed method was 8, and there are five S-boxes with this lowest DU. By way of comparison, 18 and 10 are the worst and best achievable DU scores in Reference [40]. The features of all five S-boxes having the lowest DU by the proposed method are listed in Table 11. If we change the preference criteria in the proposed method to update the *S_G_* S-box from nonlinearity to differential uniformity, then the best obtainable S-box is S3 listed in Table 11. It is worth noting that the features of S-box S3 when compared with existing ones listed in Table 8 are cryptographically better than most of the S-boxes of Table 8. For the SAC criteria, there were 1717 S-boxes which have a SAC equal to the ideal value that is exactly 0.5. Our worst, best, and average SAC was 0.5249, 0.5, and 0.5019, respectively, which was slightly better than respective scores of 0.4832, 0.5264, and 0.5020, respectively, in Reference [40]. The number of S-boxes satisfying 0.495 ≤ SAC ≤ 0.505 is 62677. The worst, best, and average BIC-nonlinearity values were 100.5, 105.57, and 103.53, respectively. There exist more than 56% of all S-boxes whose BIC-Nonlinearity was higher than 103.5, and more than 21% with a BIC-NL above 104. Regarding BIC-SAC, the worst, best, and average scores are 0.5135, 0.5, and 0.5019, respectively, and as much as 85.7% of all S-boxes had a BIC-SAC in [0.495, 0.505]. Thereby, all generated S-boxes showed good satisfaction of the BIC property.

Thus, it is evident that the batch-generation capability of the proposed method for bijective S-boxes construction is commendable as it satisfies cryptographic properties reasonably well under different cases and conditions.

### 4.8. Performance of Small-Sized S-Boxes

In addition, we investigated the proposed method for the construction of small-sized bijective S-boxes of sizes 4 × 4, 5 × 5, 6 × 6, and 7 × 7. Different small-sized S-boxes obtained with the proposed method for different *n* are provided in Table 12. Their nonlinearity scores and differential uniformities are listed in Table 13. The generic methods that can synthesize S-boxes of varied small sizes are rarely investigated. In the literature, there exist optimization-based methods for the synthesis of bijective S-boxes for 5 ≤ *n* ≤ 8. In 1998, one such approach was suggested by Millan, which was based on a hill climbing technique for the evolution of S-boxes [53]. Fuller et al. applied a heuristic technique to optimize the power mapping-based S-boxes through some iterated mutation operations suggested by him [54]. In Reference [55], Laskari et al*.* adopted a particle swarm optimization and differential evolution techniques to obtain a number of optimized bijective S-boxes. Tesar designed a special genetic algorithm with a total tree search to evolve small-sized S-boxes in Reference [56]. Of late, Picek et al. has designed a new cost function for evolving S-boxes and different evolutionary techniques, such as genetic algorithm (GA), genetic with tree search (GaT), and local search algorithm (LSA), were analyzed for evolving S-boxes with a new cost function [30]. To justify the improved performance of our method, results were compared with these existing methods in Table 14. We reported the best results for all methods. It is clear from Table 14 that the proposed S-boxes had a significantly higher nonlinearity than existing methods for all sizes. The comparison verified the better performance of our proposed method for the construction of small-sized bijective S-boxes as well.

## 5. Conclusions

This paper reports a design procedure for cryptographic substitution-boxes using a hyper-chaotic system. Firstly, a new five-dimensional hyperchaotic system was modeled, which holds some merits over the existing systems. Then, the new hyper-chaotic system was utilized to propose a method for bijective S-box construction. The anticipated method systematically searched the best possible S-box for a given size on the basis of nonlinearity by exploiting the dynamics of new hyperchaotic system. Some standard performance criteria were applied to assess the security strength of the proposed S-box method. The obtained results were compared with some recent S-box proposals to justify the upright performance of the proposed method. The effectiveness of the batch-generation capability of our method was analyzed statistically. It showed that it was possible to obtain 8 × 8 S-boxes with a differential uniformity of 8. Additionally, the same method was also investigated to yield small-sized S-boxes. It has been shown that our method was competent enough to yield better nonlinear small-sized S-boxes. To the best of our knowledge, this is first chaos-based method that attempts to synthesis small-sized S-boxes. The S-box construction method is key-dependent and a large number of strong S-boxes can be obtained with a minute change of any of the key components. The proposed method for S-box generation satisfied all the criteria of a strong S-box very well, and the constructed S-boxes were suited for usage in a strong block cipher design and other security applications.

## Figures and Tables

**Table 1:** Comparison of high dimensional chaotic/hyperchaotic systems adopted for 8 × 8 S-box construction.

S-Box Method	Type of Chaos	Dimension	*LE*	*D_KY_*
Ref. [19]	Hyperchaotic	4-D	0.0905	2.0529
Ref. [20,21]	Lorenz chaotic	3-D	0.906	2.062
Ref. [22]	fractional Rössler chaotic	3-D	NR	NR
Ref. [23]	Chaotic	3-D	0.064	2.05
Ref. [24]	fractional Chen chaotic	3-D	0.0119	NR
Ref. [25]	Chaotic	3-D	NR	NR
Ref. [21,29]	Rössler chaotic	3-D	0.0714	2.0132
Proposed	Hyperchaotic	5-D	4.90182	3.334

**Table 2:** Proposed 8 × 8 Substitution-box.

160	176	224	194	124	25	15	158	234	200	236	220	81	238	173	155
149	31	94	199	55	57	110	23	40	18	174	117	11	196	135	221
175	205	82	125	203	212	241	109	139	76	206	43	148	195	126	129
248	159	28	20	187	223	213	33	231	165	197	45	182	120	192	116
63	36	133	106	100	145	216	214	243	21	7	8	204	5	210	68
89	48	153	178	14	147	103	41	143	115	232	46	172	237	93	167
12	180	70	202	107	80	29	251	75	42	71	131	235	72	101	19
146	138	222	34	161	84	104	186	85	122	229	38	166	118	190	53
171	230	67	113	69	51	96	6	111	156	150	32	54	123	255	183
245	121	10	188	209	211	127	177	169	250	86	228	52	92	47	3
218	144	17	154	170	142	9	132	157	112	65	1	225	249	73	163
59	219	254	27	191	207	189	95	130	181	2	141	61	0	246	50
226	227	22	128	62	201	151	91	39	77	102	253	98	66	108	49
215	152	105	30	247	239	24	88	78	60	136	114	26	56	64	119
198	179	44	13	97	185	140	35	58	244	4	90	87	79	83	208
37	242	134	168	162	240	184	74	99	193	16	164	233	137	217	252

**Table 3:** Nonlinearities of the component Boolean functions in the proposed S-box.

*g*_8_	*g*_7_	*g*_6_	*g*_5_	*g*_4_	*g*_3_	*g*_2_	*g*_1_
108	110	108	108	106	110	108	110

**Table 4:** Dependency matrix for SAC.

0.4687	0.5312	0.5312	0.5312	0.4062	0.5	0.5312	0.4531
0.5937	0.5625	0.5	0.5	0.5	0.5156	0.4687	0.5468
0.5625	0.5312	0.5312	0.5	0.5156	0.5	0.4531	0.4843
0.4687	0.5	0.4843	0.4843	0.5156	0.5156	0.5937	0.4843
0.4218	0.5625	0.5156	0.4843	0.4531	0.5312	0.5468	0.4843
0.4062	0.5625	0.4843	0.5781	0.4687	0.5	0.5312	0.5
0.5156	0.48437	0.5	0.4687	0.4843	0.4687	0.4375	0.4531
0.4843	0.5	0.5312	0.5156	0.5156	0.4531	0.5312	0.4687

**Table 5:** BIC results for nonlinearity.

	*g*_1_	*g*_2_	*g*_3_	*g*_4_	*g*_5_	*g*_6_	*g*_7_	*g*_8_
*g*_1_	-	104	104	104	106	102	106	104
*g*_2_	104	-	104	106	104	104	102	104
*g*_3_	104	104	-	102	102	106	100	104
*g*_4_	104	106	102	-	106	102	106	102
*g*_5_	106	104	102	106	-	100	108	104
*g*_6_	102	104	106	102	100	-	106	102
*g*_7_	106	102	100	106	108	106	-	108
*g*_8_	104	104	104	102	104	102	108	-

**Table 6:** BIC results for the SAC.

	*g*_1_	*g*_2_	*g*_3_	*g*_4_	*g*_5_	*g*_6_	*g*_7_	*g*_8_
*g*_1_	-	0.4960	0.4765	0.4980	0.5175	0.5058	0.4707	0.4980
*g*_2_	0.4960	-	0.4726	0.5	0.5273	0.4863	0.5332	0.4843
*g*_3_	0.4765	0.4726	-	0.5156	0.4726	0.5019	0.5058	0.4960
*g*_4_	0.4980	0.5	0.5156	-	0.4863	0.5390	0.5175	0.4980
*g*_5_	0.5175	0.5273	0.4726	0.4863	-	0.4824	0.5078	0.5019
*g*_6_	0.5058	0.4863	0.5019	0.5390	0.4824	-	0.5097	0.4902
*g*_7_	0.4707	0.5332	0.5058	0.5175	0.5078	0.5097	-	0.5253
*g*_8_	0.4980	0.4843	0.4960	0.4980	0.5019	0.4902	0.5253	-

**Table 7:** Differential matrix for DU.

6	6	6	8	6	6	8	6	10	4	8	8	8	6	8	8
6	6	8	10	6	8	8	6	6	6	8	6	8	6	6	6
4	8	8	8	8	6	6	6	8	8	6	6	6	6	6	6
6	6	8	8	8	6	6	8	6	6	6	6	8	8	6	6
10	8	6	6	6	6	6	6	6	6	8	6	6	6	6	6
8	6	6	8	6	6	8	6	8	6	8	6	6	6	6	8
6	6	6	8	8	6	8	6	6	6	6	6	6	6	8	8
8	8	8	8	6	8	8	4	6	6	6	8	6	6	8	6
6	6	6	6	6	8	6	6	6	8	8	8	6	6	6	6
6	6	8	6	6	6	6	6	8	6	6	6	8	6	6	6
8	6	6	8	8	6	8	6	8	8	6	6	6	6	6	8
8	8	6	8	6	6	6	6	8	6	6	6	6	8	6	8
8	6	8	8	6	6	8	8	8	6	6	6	6	8	8	8
8	6	8	6	8	6	6	6	8	8	6	6	8	6	6	6
8	8	8	6	8	6	6	8	8	6	6	6	10	6	6	6
8	6	8	6	6	6	8	6	6	6	6	8	8	8	6	-

**Table 8:** Comparison of nonlinearity, SAC, and BIC scores of recent 8 × 8 S-boxes.

S-Box Method	Nonlinearity			SAC	BIC-SAC	BIC-NL	DU
	*nl_min_*	*nl_max_*	*nl_avg_*				
Proposed	106	110	108.5	0.5017	0.5026	104	10
Ref. [19]	102	108	106	0.5002	0.5013	104.4	10
Ref. [20]	100	106	103.2	0.5048	0.5009	103.7	10
Ref. [21]	98	108	103	0.5012	0.4988	104.07	12
Ref. [22]	100	108	104.5	0.4978	0.5009	103.6	12
Ref. [23]	104	108	105.80	0.4976	0.5032	104.5	10
Ref. [24]	100	108	104.7	0.4982	0.4942	103.1	10
Ref. [25]	104	110	106	0.5039	0.5058	103.38	10
Ref. [29]	102	108	105.25	0.4985	0.4985	103.7	12
Ref. [33]	98	110	105.5	0.4937	0.5013	105.7	32
Ref. [39]	102	108	105.25	0.4956	0.4996	103.8	10
Ref. [40]	106	108	106.7	0.4941	0.4957	103.5	10
Ref. [41]	106	108	107.25	0.5034	0.4980	105.29	12
Ref. [42]	99	106	103.5	0.5066	0.5029	103.35	12
Ref. [43]	106	110	107	0.5014	0.5016	104.21	10
Ref. [44]	104	108	106.75	0.5031	0.5074	103.64	12
Ref. [45]	96	106	103.25	0.5151	0.4864	103.07	44
Ref. [46]	98	108	102.25	0.4836	0.4948	101.57	14
Ref. [47]	98	108	104	0.5039	0.5078	104	12
Ref. [48]	84	106	100	0.4812	0.4962	101.9	16
Ref. [49]	100	106	103	0.5020	0.4998	102.93	10
Ref. [50]	106	108	106.5	0.4978	0.5029	104.21	10
Ref. [51]	105	107	106	0.5066	0.5065	103	12
Ref. [52]	106	108	107.5	0.4943	0.4982	104.36	10

**Table 9:** Statistics of 100,000 generated 8 × 8 S-boxes.

Parameter	Worst	Best	Average
Average Nonlinearity	102	108.5	104.665
DU	18	8	-
SAC	0.5249	0.5	0.5019
BIC-Nonlinearity	100.5	105.57	103.53
BIC-SAC	0.5135	0.5	0.5019

**Table 10:** Analysis of 100,000 generated 8 × 8 S-boxes under some conditions.

Condition	Percentage of S-Boxes
average Nonlinearity ≥ 104	90.932
average Nonlinearity ≥ 105	35.467
DU ≤ 10	40.871
0.495 ≤ SAC ≤ 0.505	62.677
BIC- Nonlinearity ≥ 103.5	56.109
BIC- Nonlinearity ≥ 104	21.549
0.495 ≤ BIC-SAC ≤ 0.505	85.7

**Table 11:** Features 8 × 8 S-boxes having differential uniformity equals to 8.

#	DU	Average NL	SAC	BIC-NL	BIC-SAC
S1	8	105	0.4963	104.21	0.5006
S2	8	104.5	0.4963	103.64	0.5015
S3	8	105.75	0.5017	103.57	0.4990
S4	8	104.25	0.4976	104.36	0.5035
S5	8	105	0.5061	102.36	0.5015

**Table 12:** Proposed small-sized bijective S-boxes.

**4 × 4 S-Box**															
11				9				4				2			
10				3				7				14			
1				12				5				0			
13				6				8				15			
**5 × 5 S-box**															
3		12		30		28		15		27		11		25	
14		31		23		8		5		7		4		1	
29		10		0		16		19		26		2		21	
18		24		9		20		17		13		22		6	
**6 × 6 S-box**															
60		48		62		21		61		4		54		45	
46		20		11		55		25		16		9		57	
31		39		12		26		6		24		19		44	
13		63		23		52		0		37		33		35	
17		10		41		1		36		18		51		58	
42		8		38		5		40		2		14		59	
43		27		28		15		53		32		56		3	
50		22		29		34		30		7		49		47	
**7 × 7 S-box**															
125	88	54	109	1	86	64	115	0	27	106	13	56	8	42	65
100	92	28	23	61	117	30	96	73	49	32	122	98	80	76	43
123	59	47	70	12	6	22	116	10	118	31	101	50	114	33	52
84	14	48	113	26	67	46	58	75	17	69	29	79	82	7	35
83	112	45	110	51	16	53	5	107	57	121	127	102	36	93	40
71	68	2	95	21	62	89	38	15	44	94	9	20	37	124	119
41	4	19	97	66	24	39	120	99	60	25	72	55	11	108	126
63	77	85	105	81	103	91	90	18	111	74	87	3	78	104	34

**Table 13:** Nonlinearity of the component functions and differential uniformity of the proposed small-sized S-boxes in Table 12.

S-Box	Nonlinearities							DU
	*nl_g_*_1_	*nl_g_*_2_	*nl_g_*_3_	*nl_g_*_4_	*nl_g_*_5_	*nl_g_*_6_	*nl_g_*_7_	
4 × 4	4	4	4	4				4
5 × 5	10	12	12	12	10			6
6 × 6	22	24	24	24	24	24		6
7 × 7	52	52	52	52	50	48	52	8

**Table 14:** Comparison of the average nonlinearities of the small-sized S-boxes.

S-box	Ref. [30]	Ref. [53]	Ref. [54]	Ref. [55]	Ref. [56]	Proposed
4 × 4	4	-	-	-	-	4
5 × 5	10	10	6	10	10	11.2
6 × 6	22	20	18	22	22	23.67
7 × 7	48	46	42	48	48	51.14
8 × 8	104	102	104	98	104	108.5
